# Multimodal Echocardiography for Diagnostic Value of Type 2 Diabetes Mellitus Complicated with Left Anterior Descending Artery Stenosis: A Retrospective Case-Control Study

**DOI:** 10.2174/0115734056410845251205082610

**Published:** 2026-01-15

**Authors:** Zhiyong Li, Jingwu Wu, Jie You, Zhen He, Liye Wang, Xiaoni Zhou, Gang Hu

**Affiliations:** 1 Department of Ultrasound, the Second People's Hospital of Jingdezhen, 333000 Jingdezhen, Jiangxi, China; 2 Second Clinical Medical College, Second Affiliated Hospital of Xi'an Jiaotong University, 710004 Xi'an, Shaanxi, China; 3 National Engineering Research Center for Bioengineering Drugs and the Technologies, Institute of Translational Medicine, Jiangxi Medical College, Nanchang University, Nanchang, 330031, Jiangxi, China; 4 Nanchang University Queen Mary School, 330031 Nanchang, Jiangxi, China; 5 Department of Obstetrics & Gynecology, The First Affiliated Hospital, Jiangxi Medical College, Nanchang University, 330006 Nanchang, Jiangxi, China; 6 Department of Obstetrics & Gynecology, The Second People's Hospital of Jingdezhen, 333000 Jingdezhen, Jiangxi, China; 7 School of Life Sciences, Nanchang University, 330031 Nanchang, Jiangxi, China; 8 Clinical laboratory, the Second People's Hospital of Jingdezhen, Key Laboratory of Jingdezhen for Cellular and Molecular Medicine, 333000 Jingdezhen, Jiangxi, China

**Keywords:** Type 2 diabetes mellitus, Coronary artery disease, Multimodal echocardiography, Two-dimensional echocardiography, Speckle tracking echocardiography

## Abstract

**Introduction:**

Type 2 diabetes mellitus (T2DM) significantly increases the risk of coronary heart disease (CHD), with left anterior descending artery (LAD) stenosis being a critical determinant of prognosis. While coronary angiography (CAG) and coronary computed tomography angiography (CCTA) are standard diagnostic tools, they have inherent limitations. This study aimed to evaluate the clinical value of multimodal echocardiography in assessing LAD stenosis severity in patients with T2DM.

**Methods:**

In this retrospective case-control study, 96 T2DM patients with LAD stenosis ≥50% (by CAG) and 96 with <50% stenosis were consecutively enrolled. All participants underwent two-dimensional echocardiography (2DE), two-dimensional speckle tracking echocardiography (2D-STE), and coronary artery ultrasound imaging (CA-USI). Diagnostic performance was compared with CAG as the reference standard.

**Results:**

2D-STE and CA-USI demonstrated superior diagnostic performance for LAD stenosis compared to 2DE. Specifically, 2D-STE yielded an area under the curve (AUC) of 0.818, sensitivity of 0.760, and specificity of 0.875; CA-USI showed an AUC of 0.849, sensitivity of 0.802, and specificity of 0.895; while 2DE had an AUC of 0.583, sensitivity of 0.239, and specificity of 0.927. Group differences in regional wall motion abnormality, LAD plaque, global longitudinal strain, and peak diastolic velocity were all significant (*P*<0.05).

**Discussion:**

These findings indicated that 2D-STE and CA-USI outperformed conventional 2DE in detecting LAD stenosis among T2DM patients, providing more comprehensive functional and structural insights. The integration of strain imaging and coronary ultrasound enables earlier detection of subclinical myocardial impairment and plaque burden, offering practical value for risk stratification and longitudinal follow-up in diabetic populations. Compared with prior single-modality echocardiographic assessments, the multimodal approach in this study enhances diagnostic confidence and may reduce reliance on invasive CAG for preliminary evaluation. However, as a retrospective single-center analysis, potential selection bias and the modest sample size may limit generalizability. Future multicenter prospective trials are warranted to validate these findings and explore the incorporation of artificial intelligence-assisted analysis to improve precision and reproducibility.

**Conclusion:**

Multimodal echocardiography, especially 2D-STE and CA-USI, provides a more accurate assessment of LAD stenosis in T2DM patients than conventional 2DE. Specifically, for detecting LAD stenosis ≥50%, 2D-STE achieved an AUC of 0.818, sensitivity of 0.760, and specificity of 0.875; CA-USI yielded an AUC of 0.849, sensitivity of 0.802, and specificity of 0.895; while 2DE had an AUC of 0.583, sensitivity of 0.239, and specificity of 0.927. These findings support the clinical utility of 2D-STE and CA-USI for comprehensive coronary evaluation in patients with T2DM.

## 1 INTRODUCTION

Type 2 diabetes mellitus (T2DM) is a highly prevalent chronic disease, accounting for over 90% of all diabetes cases [1]. Patients with T2DM are at a significantly increased risk of developing coronary heart disease (CHD), with the comorbidity rate rising annually. Cardiovascular diseases remain the leading cause of mortality in T2DM patients, contributing to approximately 75% of deaths [2-4]. Therefore, timely and accurate assessment of CHD in T2DM patients is crucial for guiding clinical management and mitigating adverse cardiovascular events.

The left anterior descending artery (LAD) is the primary coronary vessel implicated in CHD. Currently, coronary angiography (CAG) and coronary computed tomography angiography (CCTA) are the standard techniques for evaluating LAD stenosis and its severity [5-7]. Although these methods are highly effective, they present notable limitations, including radiation exposure, invasiveness, and high costs, which restrict their widespread application in T2DM screening. In contrast, echocardiography offers significant advantages. It is non-invasive, cost-effective, and sufficiently accurate, making it particularly suitable for large-scale screening [8, 9].

The field of ultrasound imaging has increasingly adopted multimodal echocardiography, an approach that integrates multiple echocardiographic techniques to achieve a more comprehensive, precise, and sensitive assessment [10, 11]. This approach has been applied across various cardiac conditions. For example, in heart failure, multimodal echocardiography facilitates the simultaneous evaluation of ventricular function, valvular integrity, and myocardial abnormalities. In congenital heart disease, it enables detailed visualization of complex anatomical structures and shunt dynamics [12, 13]. Furthermore, in hypertensive heart disease, it provides critical insights into left ventricular hypertrophy and diastolic dysfunction.

In the specific context of T2DM-associated CHD, multimodal echocardiography offers several potential advantages. It enables a more holistic assessment of cardiac structure and function, which is essential for early diagnosis and disease monitoring [14]. Additionally, it provides functional insights beyond mere anatomical visualization, enhancing the understanding of myocardial performance. Its non-invasive nature makes it well-suited for repeated assessments, which is particularly beneficial for long-term disease management [15, 16]. Moreover, its cost-effectivenesscompared to other imaging modalities could facilitate more frequent monitoring, ultimately improving patient outcomes. Despite these potential benefits, most assessments of CHD in T2DM patients continue to rely primarily on laboratory parameters, with limited studies evaluating the utility of multimodal echocardiography in this population [17].

This study adopts a multimodal echocardiographic approach, incorporating two-dimensional echocardiography (2DE), two-dimensional speckle tracking echocardiography (2D-STE), and coronary artery ultrasound imaging (CA-USI) to assess T2DM patients. Using CAG as the gold standard, this study aims to evaluate the clinical value of multimodal echocardiography in detecting LAD stenosis and determining its severity in T2DM patients.

## 2 MATERIALS AND METHODS

### 2.1 General Information

This study was approved by the Ethics Committee of the Second People's Hospital of Jingdezhen (Approval No. 2025-LLLW-09), with all patients and their families providing informed consent. From January 2023 to December 2024, 96 T2DM patients diagnosed with LAD stenosis ≥50% *via* CAG were selected as the experimental group (Moderate LAD stenosis: 50–70% by CAG; Severe LAD stenosis: >70% by CAG). Another 96 T2DM patients with LAD stenosis <50% were chosen as the control group following a 1:1 random matching principle. Sample size was based on patient availability during the study period. All patients and controls were enrolled consecutively from the pool of patients undergoing coronary angiography. Any incomplete records were excluded prior to analysis. After exclusion, the final analytic dataset contained no missing values for key variables. No sensitivity analysis was performed in this study.

### 2.2 Inclusion and Exclusion Criteria

#### 2.2.1 Inclusion Criteria

The inclusion criteria were as follows: patients aged 40–80 years with a confirmed diagnosis of T2DM according to the American Diabetes Association (ADA) or World Health Organization (WHO) criteria; presence of clinical indications for CAG, including at least one of the following: (1) typical angina or angina-equivalent symptoms (*e.g*., exertional chest pain or dyspnoea); (2) abnormal findings on resting or exercise electrocardiogram (ECG); (3) positive results on non-invasive cardiac stress tests (such as treadmill exercise test, stress echocardiography, or myocardial perfusion imaging); or (4) high cardiovascular risk profile as determined by the treating physician. Only patients who subsequently underwent CAG and were found to have either LAD artery stenosis ≥50% (observation group) or <50% (control group) were included. Additional inclusion criteria were: stable blood pressure controlled below 140/90 mmHg at the time of examination; left ventricular ejection fraction (LVEF) ≥50% on echocardiography; sinus rhythm as determined by ECG; no recent acute cardiovascular events within the preceding three months; and availability of complete clinical, laboratory, and imaging data. All patients provided written informed consent.

#### 2.2.2 Exclusion Criteria

The exclusion criteria were as follows: patients with a history of myocardial infarction, cardiomyopathy, or heart failure; poor echocardiographic image quality for any modality (2DE, 2D-STE, or CA-USI), defined as the inability to obtain diagnostically adequate images after two attempts by an experienced sonographer; high cardiac output, defined as cardiac output greater than 8 L/min as measured by echocardiography and/or clinical evidence of hyperdynamic circulation (such as in the context of hyperthyroidism or significant anemia); a diagnosis of hyperthyroidism or hypothyroidism; moderate-to-severe anemia (haemoglobin <90 g/L); significant electrolyte imbalances; persistent tachycardia (resting heart rate exceeding 120 beats per minute) not controlled by medical therapy; any documented arrhythmia or non-sinus rhythm, including atrial fibrillation, atrial flutter, frequent premature ventricular contractions, or other sustained supraventricular or ventricular arrhythmias, as confirmed by ECG and specialist review; a history of percutaneous coronary intervention or coronary artery bypass grafting; significant valvular heart disease (moderate or severe stenosis or regurgitation); congenital heart disease or other structural cardiac abnormalities; severe chronic pulmonary disease or pulmonary hypertension (mean pulmonary artery pressure >25 mmHg on echocardiography); uncontrolled hypertension (resting blood pressure ≥160/100 mmHg) or hypotension (systolic blood pressure <90 mmHg); chronic kidney disease stage 4–5 (estimated glomerular filtration rate <30 mL/min/1.73 m^2^); significant hepatic dysfunction (Child-Pugh class B or C); active infection or systemic inflammatory disease; ongoing malignancy or recent cancer treatment within the previous six months; pregnancy or lactation; incomplete clinical data or missing laboratory or imaging records; or unwillingness or inability to participate due to factors, such as severe cognitive impairment, psychiatric illness, or withdrawal of consent. All exclusion criteria were rigorously applied to ensure the reliability and validity of the study cohort.

### 2.3 Instruments and Testing Methods

A PHILIPS EPIQ7C and a Siemens ACUSON SC2000 color Doppler echocardiograph equipped with two-dimensional speckle-tracking quantitative analysis software were used. All imaging procedures strictly followed the recommendations of the American Society of Echocardiography (ASE) and the European Association of Cardiovascular Imaging (EACVI) [18]. Patients underwent multimodal echocardiography (2DE, 2D-STE, and CA-USI), with all examinations performed in the left lateral position under ECG monitoring.

Two-dimensional echocardiography (2DE) was used to obtain multi-view cardiac images to assess regional wall motion and measure the left ventricular end-diastolic diameter (LVEDD), left ventricular ejection fraction (LVEF), interventricular septal end-diastolic thickness (IVSEDT), and left atrial systolic diameter (LASD). Two-dimensional speckle-tracking echocardiography (2D-STE) involved dynamic acquisition of three consecutive cardiac cycles in apical four-chamber, two-chamber, and long-axis views. The software then quantified left ventricular global longitudinal strain (LVGLS) and the longitudinal peak strain (LPS) of the septal and anterior walls. Coronary artery ultrasonography (CA-USI) was performed by scanning along the anatomical course of the left anterior descending artery (LAD) to document plaque characteristics and measure peak diastolic velocity (PDV). To minimize bias, standardized protocols were applied, and all echocardiograms were interpreted by trained physicians blinded to CAG results.

Prior to enrollment, an a priori sample size calculation was conducted based on preliminary data for the primary diagnostic endpoint (*e.g*., sensitivity or AUC of 2D-STE and CA-USI for detecting ≥50% LAD stenosis). With a significance level of 0.05, statistical power of 80%, and an anticipated difference in diagnostic performance between modalities, a minimum of 92 patients per group was required. The final sample size of 96 patients per group was therefore considered adequate for detecting clinically meaningful differences. To evaluate the CA-USI imaging success rate, all initially screened cases were reviewed. Of 287 patients assessed, 24 (8.4%) were excluded due to suboptimal CA-USI image quality, yielding a success rate of 91.6%. Similar image-quality criteria were applied to 2DE and 2D-STE. Only patients with diagnostically adequate images across all three modalities were included in the final analysis. Inter- and intra-observer variability was assessed by two experienced, blinded echocardiographers analyzing randomly selected cases, demonstrating strong agreement and high reproducibility.

Regional wall motion abnormalities (RWMA) were defined according to ASE criteria as any myocardial segment with an abnormal contraction pattern on 2DE or 2D-STE. The LAD plaque ratio was defined as the proportion of patients with visibly detectable atherosclerotic plaque within the LAD territory on CA-USI.

For 2DE, RWMA in the LAD territory was assessed using standard apical and parasternal views, with any abnormality considered a positive result. For 2D-STE, global longitudinal strain (GLS) and segmental LPS of the septum and anterior wall were evaluated; values below their established cutoff thresholds (*e.g*., GLS <18%) were considered indicative of significant LAD stenosis. For CA-USI, the presence of plaque and/or a PDV above the ROC-derived threshold was defined as diagnostic of significant stenosis.

The sensitivity, specificity, and diagnostic accuracy of each modality were calculated using CAG-confirmed LAD stenosis (≥50%) as the gold standard. Only patients with high-quality, interpretable imaging across all modalities were included in the final analysis.

### 2.4 Statistical Analysis

SPSS 26.0 was used for statistical analysis. Continuous variables are presented as mean ± SD and analyzed using independent t-tests, while categorical variables are shown as n (%) and compared using χ^2^ tests. Sensitivity, specificity, accuracy, positive predictive value, and negative predictive value of 2DE, 2D-STE, and CA-USI for LAD stenosis ≥50% were calculated using CAG as the gold standard. All echocardiographic parameters were analyzed as continuous variables; Continuous variables were analyzed as continuous measures; for diagnostic performance analyses, prespecified/ROC-derived cutoffs were applied. Agreement was assessed *via* the Kappa test (Kappa ≤0.4: slight; 0.41-0.6: moderate; ≥0.61: substantial). ROC curves were plotted, and AUC was calculated. For each imaging technique, sensitivity, specificity, and accuracy were calculated based on defined thresholds for key parameters (*e.g*., LVGLS cutoff, presence of RWMA, presence of LAD plaque on CA-USI), with reference to published diagnostic criteria. Statistical significance was set at *P* <0.05.

## 3 RESULTS

### 3.1 Flow Diagram and Comparison of General Clinical Features

In this study, patients with T2DM were assessed for eligibility for inclusion in the final analysis. A total of 287 patients were initially assessed. However, 69 patients were excluded because they did not meet the inclusion criteria or because they met the exclusion criteria. Specifically, 31 patients were excluded due to incomplete data, and 24 were excluded due to poor imaging quality (including 10 for suboptimal 2DE, 8 for 2D-STE, and 6 for CA-USI). After careful screening and exclusion of patients with incomplete information, 96 patients were ultimately included in both the observation and control groups, in accordance with a 1:1 allocation ratio. A detailed overview of the patient selection process and the reasons for exclusion is presented in Fig. (**1**). Analysis of the baseline characteristics revealed no significant differences between the two groups in terms of gender, age, BMI, comorbidities, blood pressure, heart rate, HDL-C, baseline glycemia, glycate hemoglobin, or the use of insulin and metformin. However, significant differences were observed in T2DM duration, LDL-C, triglyceride levels, and the use of statins and antiplatelet agents, as detailed in Table **1**.

### 3.2 Comparison of Multimodal Echocardiographic Parameters between the Observation and Control Groups

**Fig. (2):**
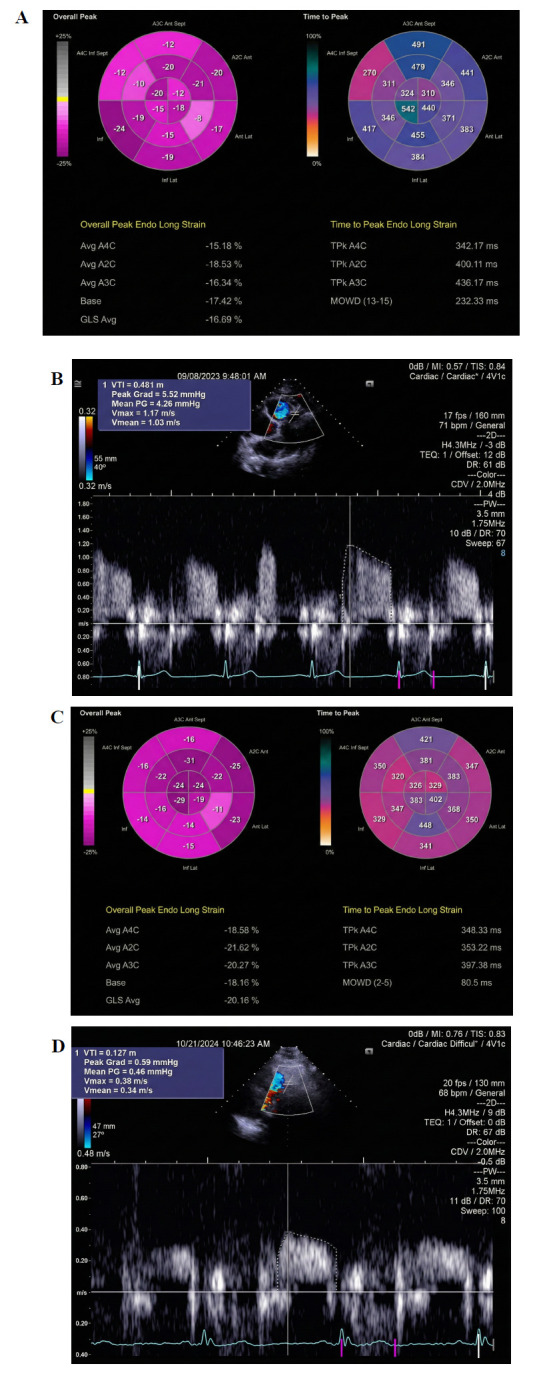
The presentation of 2D-STE and CA-USI plots for both patient groups. **(A)**, 2D-STE bull's-eye plot of the observation group. **(B)**, CA-USI plot of the same patient in the observation group. **(C)**, 2D-STE bull's-eye plot of the control group. **(D)**, CA-USI plot of the same patient in the control group. 2D-STE, two-dimensional speckle tracking echocardiography. CA-USI, coronary artery ultrasound imaging.

No statistically significant differences were observed in LVEDD, IVSEDT, LASD, or LVEF between the observation and control groups (*P*>0.05). However, RWMA and the LAD plaque ratio were significantly higher in the observation group than in the control group (*P*<0.05). Conversely, LVGLS, septal LPS, and anterior wall LPS were significantly lower in the observation group compared to the control group (*P*<0.05). Additionally, PDV was significantly elevated in the observation group relative to the control group (*P*<0.05) (Table **2** and Fig. **2**).

### 3.3 Comparison of Multimodal Echocardiographic Parameters between the Moderate and Severe Stenosis Subgroups

No statistically significant differences were observed in LVEDD, IVSEDT, LASD, or LVEF between the moderate and severe stenosis subgroups (*P*>0.05). However, RWMA and the LAD plaque ratio were significantly lower in the moderate stenosis subgroup compared to the severe stenosis subgroup (*P*<0.05). Conversely, LVGLS, septal LPS, and anterior wall LPS were significantly higher in the moderate stenosis subgroup than in the severe stenosis subgroup (*P*<0.05). PDV was also significantly lower in the moderate stenosis subgroup than in the severe stenosis subgroup (*P*<0.05) (Table **3**).

### 3.4 Diagnostic Efficacy Analysis of Different Echocardiographic Techniques

**Fig. (3):**
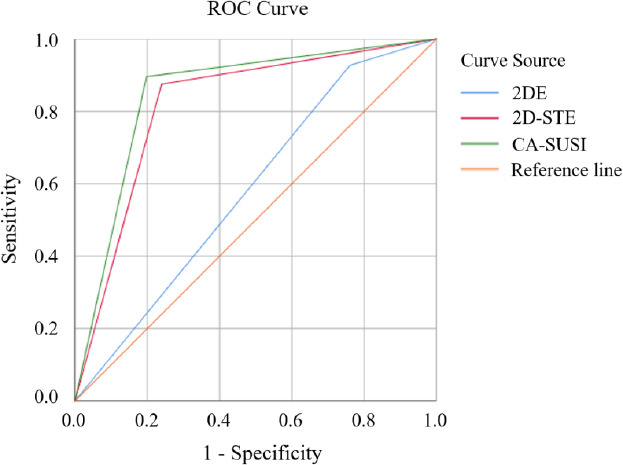
ROC curves for different echocardiographic techniques. **Abbreviations:** ROC, receiver operating characteristic. 2DE, two-dimensional echocardiography. 2D-STE, two-dimensional speckle tracking echocardiography. CA-USI, coronary artery ultrasound imaging.

Using CAG as the gold standard, the sensitivity, specificity, accuracy, positive predictive value, and negative predictive value of 2DE in assessing LAD stenosis ≥50% in T2DM patients were 0.239, 0.927, 0.571, 0.766, and 0.549, respectively. The corresponding values for 2D-STE were 0.760, 0.875, 0.817, 0.858, and 0.785, while those for CA-USI were 0.802, 0.895, 0.848, 0.885, and 0.819, respectively. The Kappa values indicating agreement between 2DE, 2D-STE, CA-USI, and CAG were 0.167, 0.635, and 0.698, respectively. Receiver operating characteristic (ROC) curves were plotted for each echocardiographic technique, with 1-specificity on the x-axis and sensitivity on the y-axis. The AUC values for 2DE, 2D-STE, and CA-USI were 0.583, 0.818, and 0.849, respectively (Table **4** and Fig. **3**).

## 4 DISCUSSION

This study employed a multimodal echocardiographic approach, integrating 2DE, 2D-STE, and CA-USI, to assess the diagnostic value and severity of LAD stenosis in patients with T2DM. The findings indicated that both 2D-STE and CA-USI outperformed conventional 2DE in terms of diagnostic accuracy (AUC: 0.818 and 0.849, respectively) and consistency with CAG (Kappa: 0.635 and 0.698, respectively). These results underscore the potential of 2D-STE and CA-USI as superior diagnostic tools in the evaluation of LAD stenosis in this high-risk patient cohort.

Recent studies have highlighted the advantages of multimodal imaging in the evaluation of CAD. For instance, Upton *et al*. leveraged convolutional neural networks (CNNs) to extract features from stress echocardiography, subsequently training machine learning classifiers to detect significant CAD, achieving a specificity of 92.7% and a sensitivity of 84.4% in cross-validation [19, 20]. This integration of imaging modalities significantly improved CAD detection rates, enhanced inter-observer agreement, and increased diagnostic confidence. The robust AUC and Kappa values observed in the present study further substantiate the role of multimodal echocardiography in enhancing diagnostic accuracy.

Prior research has demonstrated that diabetic patients exhibit increased coronary plaque vulnerability, characterised by a higher proportion of fibro-fatty components [21, 22]. Consistent with these findings, our study identified a significantly higher LAD plaque rate in the observation group than in the control group, reinforcing the association between diabetes and heightened plaque vulnerability. Additionally, emerging evidence suggests that low-density lipoprotein cholesterol (LDL-C) levels above the normal range exacerbate coronary atherosclerosis in T2DM, thereby augmenting plaque vulnerability and stenosis severity [23-25]. Our findings align with these observations and highlight the intricate nature of coronary lesions in diabetic patients, further underscoring the importance of multimodal imaging for their comprehensive evaluation.

Unlike previous investigations employing single imaging modalities, this study integrated 2DE, 2D-STE, and CA-USI, providing a more holistic assessment of coronary stenosis. This multimodal strategy enhanced diagnostic accuracy and yielded clinically relevant data to inform therapeutic decision-making [26, 27]. By specifically targeting T2DM patients, a population at elevated cardiovascular risk, the study provided insights into the impact of diabetes on coronary stenosis, facilitating the development of more personalised diagnostic and treatment strategies.

Future research should prioritise expanding sample sizes and conducting multicentre trials to mitigate single-centre bias and enhance external validity. Prospective studies should further confirm the diagnostic utility of 2D-STE and CA-USI in this context. Moreover, integrating artificial intelligence (AI) methodologies, such as deep learning, into the analysis of multimodal imaging data could refine diagnostic precision and efficiency [28, 29]. Exploring advanced imaging modalities, including optical coherence tomography (OCT), may also provide more detailed characterisations of coronary plaques [30, 31]. Based on the present findings, a multimodal echocardiographic strategy combining 2DE, 2D-STE, and CA-USI is recommended for routine clinical use, as it not only augments diagnostic accuracy but also equips clinicians with valuable supplementary data to guide therapeutic interventions and improve patient outcomes.

## 5 STUDY LIMITATIONS

This research has several limitations that should be acknowledged. As a retrospective case-control study, it may be subject to biases, such as incomplete data acquisition and selection bias. The relatively small sample size may restrict the generalisability of the findings, and future studies with larger cohorts are warranted to validate the diagnostic efficacy of 2D-STE and CA-USI in evaluating LAD stenosis in T2DM patients.

Additionally, restricting the study population to patients who underwent CAG introduces potential selection bias. In routine clinical practice, CAG is typically reserved for patients with suspected or established coronary artery disease based on specific clinical indications, such as angina symptoms, abnormal non-invasive test results, or high cardiovascular risk. Consequently, the cohort may not fully represent the broader T2DM population, particularly individuals with subclinical or less severe coronary disease who were not referred for invasive evaluation. This selection process could lead to an overrepresentation of patients with advanced or symptomatic coronary lesions and may limit external validity. Moreover, by including only patients who underwent CAG, those managed conservatively or diagnosed using alternative imaging modalities were excluded. Future prospective studies with broader inclusion criteria are needed to confirm the applicability of these findings to the wider T2DM population.

## CONCLUSION

This study evaluated the diagnostic utility of multimodal echocardiography in T2DM patients with LAD stenosis, demonstrating that 2D-STE and CA-USI offer superior accuracy and greater concordance with CAG. These findings not only substantiate the efficacy of multimodal imaging in the assessment of coronary artery disease but also provide novel insights into the clinical management of T2DM patients. Future research should aim to further validate these findings and explore the integration of advanced imaging technologies and analytical methodologies to enhance diagnostic precision and clinical applicability.

## Figures and Tables

**Fig. (1):**
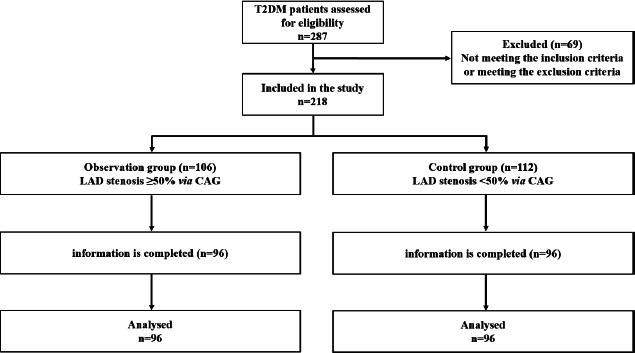
Flow diagram of the retrospective study. **Abbreviations:** T2DM, type 2 diabetes mellitus. LAD, left anterior descending artery. CAG, coronary angiography.

**Table 1:** Comparison of baseline characteristics between the two groups.

**Characteristics**		**Observation**		**Control**	***t/χ2***	***P***
		**Moderate**	**Severe**			
Gender	Male	32	20	56	0.339	0.561
	Female	24	20	40		
Age (year)		67.0 ± 3.8		66.3 ± 3.4	1.336	0.183
BMI (kg/m^2^)		22.81 ± 2.86		22.81 ± 2.61	0.009	0.993
Duration of T2DM (years)		12.2 ± 4.4		9.5 ± 4.4	4.357	<0.001
Comorbidity	Hypertension	62 (64.6%)		52 (54.2%)	2.159	0.142
	Dyslipidemia	58 (60.4%)		46 (47.9%)	3.021	0.082
	Obesity (BMI≥28)	13 (13.5%)		11 (11.5%)	0.190	0.663
Blood Pressure (mmHg)		133.6 ± 7.4/ 80.7 ± 7.4		132.5 ± 6.9/79.8 ± 7.2	1.108/0.851	0.269/0.396
Heart Rate (beats/min)		76.3 ± 8.2		75.7 ± 8.0	0.546	0.586
Lipids Assessment (mmol/L)	LDL-C	2.83 ± 0.68		2.61 ± 0.65	2.259	0.025
	HDL-C	1.06 ± 0.22		1.11 ± 0.21	1.528	0.128
	TG	2.11 ± 0.61		1.86 ± 0.56	3.113	0.002
Baseline Glycemia (mmol/L)		9.0 ± 2.7		8.4 ± 2.4	1.596	0.112
Glycate Hemoglobin (HbA1c, %)		7.9 ± 1.2		7.6 ± 1.1	1.772	0.078
Medication under Treatment	Insulin	31 (32.3%)		27 (28.1%)	0.395	0.530
	Metformin	68 (70.8%)		72 (75.0%)	0.422	0.516
	Statins	74 (77.1%)		59 (61.5%)	5.505	0.019
	Antiplatelets	88 (91.7%)		59 (61.5%)	24.41	<0.001

**Notes:** BMI, body mass index. Moderate LAD stenosis: 50–70% by CAG; Severe LAD stenosis: >70% by CAG. Antiplatelets, aspirin/clopidogrel.

**Table 2:** Baseline multimodal echocardiographic characteristics in T2DM patients with and without significant LAD stenosis.

**Parameter**	**Observation (n=96)**	**Control (n=96)**	***t/χ2***	***P***
LVEDD (mm)	46.68±3.74	45.87±3.83	1.497	0.136
IVSEDT (mm)	9.22±0.50	9.26±0.52	0.561	0.575
LASD (mm)	36.09±3.30	35.41±2.93	1.505	0.134
LVEF (%)	59.67±2.66	60.06±3.18	0.916	0.361
RWMA [n (%)]	23 (24.0)	7 (7.3)	10.114	0.001
LVGLS (%)	18.29±2.98	25.17±2.33	17.837	<0.001
Septal LAD (%)	17.79±4.55	25.51±5.61	10.476	<0.001
Anterior wall LAD (%)	16.76±3.43	26.66±3.78	19.010	<0.001
LAD plaque [n (%)]	35 (36.5)	19 (19.8)	6.596	0.010
PDV (m/s)	0.75±0.19	0.43±0.15	12.900	<0.001

**Notes:** LVEDD, left ventricular end-diastolic diameter. IVSEDT, interventricular septal end-diastolic thickness. LASD, left atrial systolic diameter. LVEF, left ventricular ejection fraction. RWMA, regional wall motion abnormality. LVGLS, left ventricular global longitudinal strain. LAD, left anterior descending artery. PDV, peak diastolic velocity.

**Table 3:** Echocardiographic markers of disease severity in moderate *versus* severe LAD stenosis among T2DM patients.

Parameter	Moderate (n=56)	Severe (n=40)	*t/χ2*	*P*
LVEDD (mm)	47.16±3.75	46.02±3.66	1.482	0.142
IVSEDT (mm)	9.21±0.50	9.25±0.51	0.361	0.719
LASD (mm)	36.56±3.47	35.43±2.96	1.665	0.099
LVEF (%)	59.32±3.17	58.56±3.33	1.147	0.254
RWMA [n (%)]	9 (16.1)	14 (35.0)	4.589	0.032
LVGLS (%)	18.61±1.91	15.22±1.46	9.421	<0.001
Septal LAD (%)	19.21±1.49	14.29±1.24	17.046	<0.001
Anterior wall LAD (%)	16.40±2.18	14.57±2.02	4.185	<0.001
LAD plaque [n (%)]	15 (26.7)	20 (50.0)	5.428	0.020
PDV (m/s)	0.62±0.09	0.90±0.11	13.470	<0.001

**Notes:** LVEDD, left ventricular end-diastolic diameter. IVSEDT, interventricular septal end-diastolic thickness. LASD, left atrial systolic diameter. LVEF, left ventricular ejection fraction. RWMA, regional wall motion abnormality. LVGLS, left ventricular global longitudinal strain. LAD, left anterior descending artery. PDV, peak diastolic velocity.

**Table 4:** Diagnostic performance of 2DE, 2D-STE, and CA-USI for detecting LAD stenosis ≥50% in T2DM patients using CAG as the reference standard.

**Method**	**AUC**	**Kappa**	**Sensitivity**	**Specificity**	**Accuracy**	**PPV**	**NPV**
2DE	0.583	0.167	0.239	0.927	0.571	0.766	0.549
2D-STE	0.818	0.635	0.760	0.875	0.817	0.858	0.785
CA-USI	0.849	0.698	0.802	0.895	0.848	0.885	0.819

**Notes:** Sensitivity, specificity, and accuracy for each echocardiographic technique were calculated as follows: For 2DE, based on the presence of regional wall motion abnormalities (RWMA) in the left anterior descending artery (LAD) territory; for 2D-STE, based on abnormal global longitudinal strain (GLS) or segmental LPS values; for CA-USI, based on the presence of LAD plaque and/or peak diastolic velocity (PDV) above the diagnostic cutoff. 2DE, two-dimensional echocardiography. 2D-STE, two-dimensional speckle tracking echocardiography. CA-USI, coronary artery ultrasound imaging. AUC: Area under the curve; PPV: Positive predictive value; NPV: Negative predictive value.

## Data Availability

The data and supportive information are available within the article.

## LIST OF ABBREVIATIONS

T2DM: Type 2 diabetes mellitus
CHD: Coronary heart disease
LAD: Left anterior descending artery
CAG: Coronary angiography
CCTA: Coronary computed tomography angiography
2DE: Two-dimensional echocardiography
2D-STE: Two-dimensional speckle tracking echocardiography
ADA: American Diabetes Association
WHO: World Health Organization
LVEF: Left ventricular ejection fraction
LVEDD: Left ventricular end-diastolic diameter
IVSEDT: Interventricular septal end-diastolic thickness
LASD: Left atrial systolic diameter

## STANDARDS OF REPORTING

STROBE guidelines were followed.
